# Determination and correction of persistent biases in quantum annealers

**DOI:** 10.1038/srep18628

**Published:** 2016-01-19

**Authors:** Alejandro Perdomo-Ortiz, Bryan O’Gorman, Joseph Fluegemann, Rupak Biswas, Vadim N. Smelyanskiy

**Affiliations:** 1 Quantum Artificial Intelligence Lab., NASA Ames Research Center, Moffett Field, CA 94035, USA; 2 University of California Santa Cruz at NASA Ames Research Center, Moffett Field, CA 94035, USA; 3 SGT Inc., 7701 Greenbelt Rd, Suite 400, Greenbelt, MD 20770, USA; 4 San Jose State Research Foundation at NASA Ames Research Center, Moffett Field, CA 94035, USA; 5 Exploration Technology Directorate, NASA Ames Research Center, Moffett Field, CA 94035; 6 Google, 150 Main St, Venice Beach, CA, 90291

## Abstract

Calibration of quantum computers is essential to the effective utilisation of their quantum resources. Specifically, the performance of quantum annealers is likely to be significantly impaired by noise in their programmable parameters, effectively misspecification of the computational problem to be solved, often resulting in spurious suboptimal solutions. We developed a strategy to determine and correct persistent, systematic biases between the actual values of the programmable parameters and their user-specified values. We applied the recalibration strategy to two D-Wave Two quantum annealers, one at NASA Ames Research Center in Moffett Field, California, and another at D-Wave Systems in Burnaby, Canada. We show that the recalibration procedure not only reduces the magnitudes of the biases in the programmable parameters but also enhances the performance of the device on a set of random benchmark instances.

Quantum annealing (QA) is a metaheuristic for solving combinatorial optimization problems^1^. The recent introduction of QA hardware by D-Wave Systems^2^,^3^ has invigorated theoretical and experimental research into the computational power and practical implementation challenges of the QA paradigm. Current research studies focus on both fundamental and applied aspects, including application to real-world problems^4^,^5^,^6^,^7^,^8^, criteria for detecting quantum speedup^9^, the computational role of quantum tunneling^10^, error-supression^11^, the relationship between classical simulated annealing and quantum annealing^12^,^13^,^14^,^15^,^16^,^17^, spin-glass perspectives on the hardness of computational problems^18^,^19^,^20^,^21^,^22^, and programming strategies that address intrinsic noise^23^,^24^.

The quantum annealers used for this study are of the second generation of D-Wave devices, also called D-Wave Two^2^: one located at NASA Ames Research Center in Moffett Field, California, (“NASA device”), and another located at D-Wave Systems in Burnaby, Canada (“Burnaby device”). These consist of 64 unit cells of a previously characterized eight-qubit unit cell^3^,^25^. In the NASA and Burnaby devices, post-fabrication characterization determined that only 509 and 424 qubits, respectively, out of the 512 qubit arrays can be reliably used for computation. The array of coupled superconducting flux qubits is, effectively, an artificial Ising spin system with programmable spin-spin couplings and longitudinal and transverse magnetic fields. It is designed to solve instances of the following (NP-hard^26^) classical optimization problem: Given a set of local fields {*h*_*i*_} 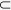 [−2, 2] and couplings {*J*_*ij*_} 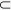 [−1, 1], find the assignment 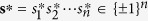, that minimizes the objective function,


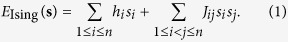


Finding the optimal s^*^ is equivalent to finding the ground state of the corresponding quantum Ising Hamiltonian 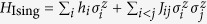, where 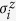 is the Pauli *z* operator acting on the *i*th spin. More details of QA can be found in the supplementary information.

Currently, D-Wave devices are only calibrated at the level of ensuring that the low-level control circuitry has its intended effect on the physical quantities like current, flux, etc., that it is meant to control^27^ (Lanting, T. Private communication, 2015). Early research into the performance of D-Wave devices has indicated the presence of significant imprecision in the setting of the fields that define the problem to be solved, a significant impairment to the successful solution of the problem^13^,^17^,^19^,^23^. Recently, some work has used a phenomenological noise model of the fields {*h*_*i*_} and {*J*_*ij*_} in which the distributions of the deviations from the programmed values are given by Gaussians with means zero and standard deviations, respectively, of 0.05 and 0.035 (in units of the maximal *J*_*ij*_), independently instantiated for each qubit and anneal and constant throughout the course of a given anneal. The parameters of the Gaussians were derived by adding in quadrature the variances of several known microscopic sources of noise (Lanting, T. Private communication, 2015). This model has been used in an attempt to explain the failure rate of D-Wave devices as partly due to misspecification of the programmable values. There are many sources of noise in quantum annealers, each with a different effect and time scale, and we address here only one manifestation. The variances we report in this paper are in a sense incomparable to those just mentioned, and relevant only within the context of the experiments described below.

The presence of systematic biases in quantum annealers has been reported elsewhere^24^. The biases referred to there are fundamentally different in nature from the ones address here, in that the former are collective biases on the qubits of ferromagnetic chains that depend on the strength and topology of the couplings therein and are due to the noise specifically caused by those couplings, and they must be determined anew for each embedding topology used. In this work, we present a methodology for determining, in parallel and using a relatively small amount of total annealing time, the persistent (see Fig. S3), systematic biases in all of the individually available programmable parameters of a quantum annealer. We show that correcting for these biases produces an increase in the quality of solutions found on a set of random benchmark instances. The strategy presented here is the first proposal for a software-level recalibration of the full device by the user, i.e. based only on the data from tailored instances and without access to the low-level control circuitry.

## Results

Because actual quantum annealers operate at non-zero temperature, there exists some threshold for the values of the fields {*h*_*i*_} and couplings {*J*_*ij*_} below which thermal effects dominate the annealing process. When the strengths of the fields and couplings are set sufficiently small, the probabilities of the final states of the qubits are well described by a Boltzmann distribution. Roughly, the relevant energy scale is given by *kT*, where *k* is Boltzmann’s constant and *T* is an effective temperature, not necessarily equal to the device temperature. (Henceforth, we will work in units in which *k* = 1.) By running experiments in this regime, persistent biases in the values of the programmed fields can be uncovered, as described in detail in the Methods section.

In our model, 
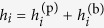
 is the effective value of the local field of qubit *i*, where 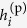 is the user-programmed value and 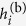 is the 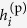-independent bias. In an ideal device, 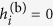.

Let 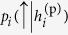 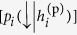 be the probability of qubit *i* being in the spin-up [spin-down] state at the end of an anneal with the programmed value 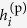. A completely thermal model for this probability is given by


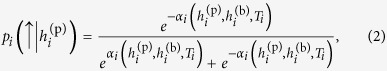


where 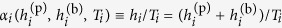 and *T*_*i*_ is the effective temperature of qubit *i*. This yields


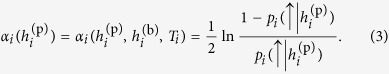


(We assume that 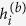 and *T*_*i*_ are constant at least over the course of the experiment.) More generally, we define the function *α*(*p*) ≡ (1/2) ln [(1 − *p*)/*p*]. Once experimental values of 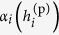 are obtained for various values of 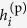, the data are fit to obtain the estimates of 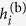 and *T*_*i*_. The biases of the couplings between qubits can be determined in a similar fashion. (See Methods section for more details).

To illustrate the efficacy of our approach in quantum annealers, we applied our method to two D-Wave devices, the NASA device and the Burnaby device.

### Determination of the *h* biases

**Figure 1:**
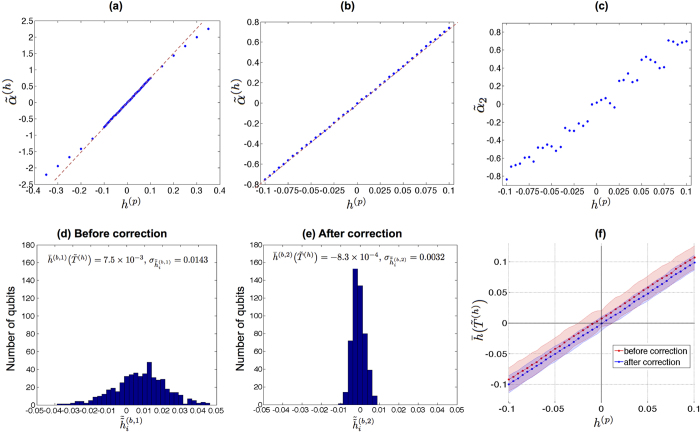
Detection and correction of systematic biases. (**a**) From a single experiment without correction, the median quantity 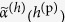, over the qubits, of the quantities 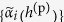 for various values of *h*^(p)^ in [−0.35, 0.35]. (**b**) Same as (**a**), but only using values of *h*^(p)^ in [−0.1, 0.1]. Note the tightness of the fit to a line, indicating the validity of the thermal model. (**c**) From the same experiment as in (**b**), the quantity 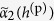 for a single, typical qubit, 2. Note the step function resulting from the limited precision of the digital-to-analog converter used to control the field. (**d**,**e**) The estimated biases 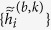 from two experiments *k* = 1, 2. The first is without correction; the second was corrected using the biases estimated from the first. Note that the distribution significantly more narrowed and centered near zero after correction. (**f**) The average over the qubits of the quantites 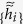 for different values of the programmed *h*^(p)^, with error bands given by the standard deviation. As for (**d**,**e**), note the narrowing and centering of the distributions.

Figure 1(a,b) shows two windows of 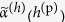 for one experiment on the NASA device. Figure 1(b) shows the range *h*^(p)^ ∈ [−0.1, 0.1] used in the calculation of the biases. There, the linearity of 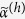, and thus the accuracy of the thermal model, is evident; this is typical of all the experiments reported. Figure 1(a) shows a wider window *h*^(p)^ ∈ [−0.35, 0.35], where the nonlinearity outside of the former range is evident, indicating the failure of the thermal model for larger magnitudes of *h*^(p)^, where annealing dynamics start to dominate thermal dynamics. Figure 1(c) shows 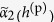, revealing the limited resolution 0.025 of the digital-to-analog converters (DACs) used to implement *h*_2_. Such stepping behavior is typical for all of the qubits.

#### Narrowing of the bias distribution

To show the correctability of the persistent biases, we ran the experiment described above repeatedly, each time attempting to correct the biases using estimates thereof from the prior iteration. Let 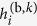 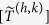 be the experimentally determined value of the bias 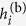 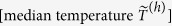 based on the *k*-iteration; by convention, set 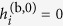. Let *h*^(p,0)^ be the desired programmed value. In the *k*-th iteration, we set


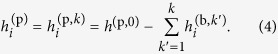


Figure 1(d,e) show the distribution of 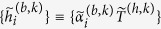 for the first and second iterations, i.e. before and after the recalibration procedure. The narrowing of the distribution is a clear indication that the procedure is working to remove the biases.

Further support for the success of recalibration is provided by looking at the distribution over the qubits of the success probabilities for all values of *h*^(p)^. Figure 1(f) shows a uniform reduction of the variance over the qubits in the values of 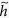. Each point is the mean 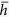 over the qubits of 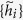 for the corresponding *h*^(p)^, and the shaded region indicates the standard deviaton. The narrowing of the distribution is clear evidence that the recalibration procedure not only narrows the distribution of the biases [Fig. 1(d,e)], as reflected in the shift of the mean, but also reduces the variance for all values of *h*^(p)^.

### Determination of the *J* biases

In the data presented here, the *J* biases were determined using (7). The programmable value of the local fields 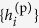 were uniformly set to zero (see Methods section for more details).

**Figure 2:**
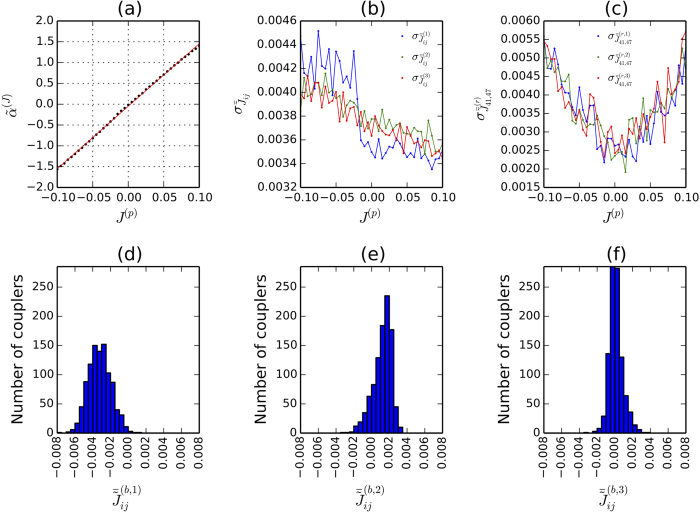
*J* biases in the Burnaby device. Data from a series of three experiments (*k* = 1, 2, 3) in which for each experiment the sums of biases estimated in the previous ones are subtracted from the original programmed values, using Eq. 5. The first experiment is without any correction, and the second and third use increasingly accurate corrections. All quantities are calculated using the mean 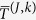 of the qubit temperatures, calculated indepently in each experiment. (**a**) From only the first experiment, the median quantity 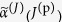, over the couplers, of the quantities 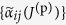 for evenly spaced values of *J*^(p)^ in [−0.1, 0.1]. (**b**) The standard deviation over the couplers of the estimated 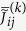 at each value of the original *J*^(*p*,0)^. (**c**) For a single, typical coupler, (41, 47), the standard deviation over 100 runs of the estimates 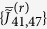 of *J*_41,47_ versus the original *J*^(*p*,0)^. (**d**–**f**) Residual biases 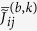 estimated from each of the experiments.

Figure 2(a) shows the median quantity 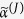 for evenly spaced values of *J*^(p)^ in [−0.1, 0.1], as well as a line fit thereto. The closeness of the fit of the line confirms the accuracy of the thermal model as for 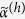.

Figure 2(b), analogous to Fig. S2(b), shows the standard deviation 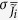 over the qubits of the estimates 
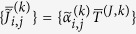
 of {*J*_*i*,*j*_} for different values of *J*^(p)^, for each of the three iterations of experiments as described above. The estimates 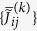 were calculated independently for each iteration *k* using its mean effective temperature 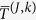. There is a significant change from the first iteration of corrections, but then the standard deviation remains approximately the same after the second iteration. Considering the average over the values of *J*^(p)^, the overall variance is about the same before and after correction, yet is much more uniform after correction, which we consider beneficial.

Figure 2(c), analogous to Fig. S1(c), shows the standard deviation 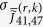 of the estimates 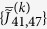. The correction seems to have no effect in this regard, and the there is a consistent increase in the variance with increasing magnitude of the programmed value *J*^(p)^. A similar phenomenon occurred in the analogous *h* data.

Define 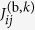 to be the estimate 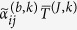 for 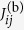 using the data from the *k*-th iteration, with 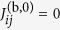 by convention. In the *k*th iteration, we set the programmed values of the couplers to


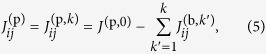


i.e. by subtracting the sums of the residual biases from the prior iterations from the desired values. Figure 2(d–f) show the narrowing of the distribution of residual *J* biases with correction.

Unlike the case for the *h* biases, for which data indicate that the distribution is essentially converged after a single iteration, here we see two new phenomena. First, the distribution continues to narrow between the second and third iterations. Second, the distribution of the residual biases from the second iteration, while narrower than that from the first, is not centered around zero. We believe this is due to overcorrection; that is, the estimates of the biases from the first iteration have a high degree of uncertainty, and so simply subtracting their values from the intended value introduces some amount of bias itself. This is consistent with the overall small magnitudes of the *J* biases relative to those of the *h* biases, especially as compared to the corresponding noise levels. This overcorrection can be mitigated by weighting the correction in a way that accounts for the uncertainty in the estimate using Bayesian reasoning. We will explore these ideas further in future work.

### Effect of correction on the performance of benchmark problems

Ultimately, the goal of calibration is to optimize the performance of a quantum annealer on problems of computational interest. It is not clear a priori that the biases present in one- and two-qubit experiments are the same as those present in anneals involving hundreds of qubits. Even if they were, their estimation would be of no practical value unless their correction improves performance. To address this, we tested the effect of correcting the *h* biases on the performance of the quantum annealer at NASA Ames, using the same parameterized random ensemble of instances used in a previous study benchmarking a D-Wave quantum annealer^9^. As in those studies, *r* is a parameter that tunes the difficulty of the average instance (the larger the *r* the more difficult the average instance). We expected the recalibration to have a major positive impact in the harder family of instances.

For each instance, the uncorrected and corrected results were compared using two methods, a “greedy” one and the elite mean. The results of the comparison are summarized in Table 1, showing the proportion of instances, for each *r*, for which the correction improved the performance, using each of the two comparison methods described above. The data set indicates that correcting for the *h* biases improves performance according to these two reasonable metrics. At a large enough range *r*, however, even correction of the biases is not enough. In this limit of large *r*, the spacing of 1/*r* between the different specifiedd *J* values is beyond the precision of the device and poorly resolved. In this limit inherent fluctuations lead to almost zero success probabilities due to problem misspecification, i.e., the device is finding the solution to another problem different from the one indicated. This would explain the possible pattern seen in the elite mean comparison (Table 1) that the advantage of correction peaks seems to peak at the level of *r* considered to correspond to the precision limit of the device. (That such a pattern is not as apparent in the greedy comparison is easily explained by natural noisiness of that comparison method, especially for instances with extremely low success probability as was the case here).

## Discussion

Disentangling the mutual effect of the *h* and *J* biases on each other by alternating between iterations of the iterations of *h* and *J* experiments (as opposed to doing each alone as reported) will likely lead to more accurate estimates of each individually. Lastly, the risk of overcorrection can be mitigated by weighting the correction by the degree of certainty of the estimate of the bias to be corrected.

Although we focused initially on a standard random ensemble of Ising instances for benchmarking the performance of quantum annealers, the effect of correcting biases should be greatest on instances whose ground states are most sensitive to misspecification of the programmable parameters^20^,^21^.

There is reason to suspect that correction will also have a beneficial effect in reducing the effect of gauge selection on success probability. While there are other suspected reasons for the effect of gauge selection (which would be non-existent in an ideal device), biases such as the ones corrected here could be one of the leading factors. The effect of gauge selection is significant, sometimes leading to an orders-of-magnitude difference in the success probabilities, and so this is a promising application for bias correction.

Importantly, while the *J* biases determined here are in general smaller than the *h* biases, numerical studies indicate that often instances are more sensitive to misspecification in the *J* parameters than in the *h* parameters^20^.

The methods presented here complement a growing suite of tools for optimal programming of quantum annealers^23^,^24^, tuning the performance thereof to cope with the intrinsic noise in current and future physical implementations.

## Methods

### Calculation of the *h* biases

To experimentally determine the biases 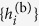, we set all 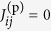 and initially assume the effect of nonzero 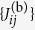 to be negligible. We therefore ran the experiments for all qubits within a given device simultaneously, with the same value of 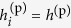 for every working qubit. Each value of *h*^(p)^ was run 100 times, where each run consisted of 1,000 annealing cycles. The probability 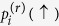 was calculated for each run *r*, and the median probability 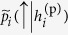 taken over the 100 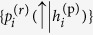 calculated. From this, we define the “median” 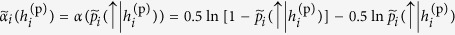. Technically, this is a slight abuse of terminology; while the median of 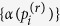 is almost the same as 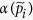 because the function *α*(*p*) is monotonic, the two quantities can differ slightly in the case of an even number of values.

Since 
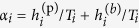
, for each qubit, we fit a line to 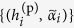 by minimizing the quadratic loss; the resulting slope gives us an estimated inverse qubit temperature 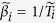, and from the intercept 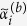 we can determine the bias in several ways.

One way is to simply use the fitted parameters as is: 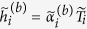. Experimental data, however, indicate that the estimates of the qubit “temperatures” calculated as above are not exactly that, but include in their calculation effects other than that due to true variation in temperature between the qubits. Some estimate of a uniform device temperature should therefore be used. (See Sec. III in the SI for more detail.) In our experiments, we used two different quantities. The first is the “mean temperature”, 
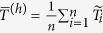
, where 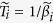 and *n* is the number of (working) qubits. The second is the “median temperature” 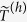, called thus not because it is the median of 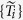 but because it is calculated by taking the inverse of the slope of the line fit to the points 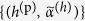, where 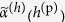 is defined as the median over the qubits of 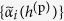. In practice, the quantities 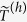 and 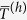 are effectively the same.

### Calculation of the *J* biases

Let 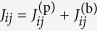 be the effective value of the coupling between qubits *i* and *j* and 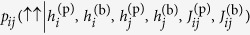 be the probability of qubits *i* and *j* both being in the spin-up state, where 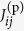 is the programmed value of the coupler and 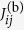 the bias, analogous to 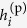 and 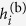, respectively. Here, we set 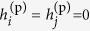, and write simply 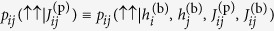. Other probabilities 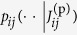, and combinations thereof such as 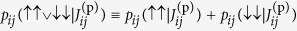, are analogously denoted.

One approach to determine the bias 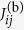 is to naively assume that 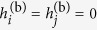, in which case the thermal distribution is modeled by


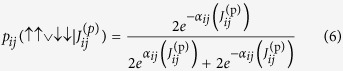


where 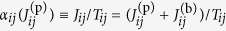. For concision, we leave the dependence of *p*_*ij*_ and *α*_*ij*_ on *h*_*i*_, *h*_*j*_, 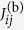, and *T*_*ij*_ implicit. Similarly to the case for *h*_*i*_, this yields


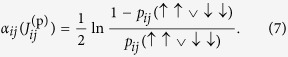


For a given value of *J*^(p)^, the experiment was run in six batches. In each batch, the programmed coupling 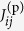 was uniformly set to *J*^(p)^ for each coupler of a pairwise disjoint subset of all of the couplers, and for each of the rest 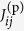 was set to zero. Over the six batches, each coupling 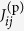 was set to *J*^(p)^ exactly once. As for the *h* biases, each value of *J*^(p)^ was run 100 times (for each coupler), with each run consisting of 1,000 annealing cycles. The median probability 
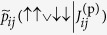
 was taken over the 100 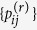 calculated from the runs, from which we calculate the “median” 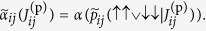 For each coupler, a line was fit to 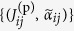, yielding a slope 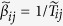 and an intercept 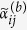. As for *h*, we define the mean temperature 
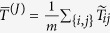
, where *m* is the number of couplers.

A more accurate estimate for 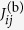 can be obtained by considering nonzero 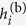 and 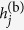 (but still setting 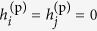). Let


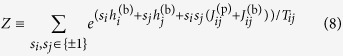


be the partition function. Then


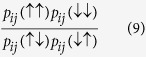



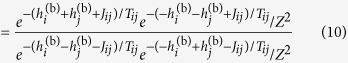



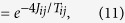


or


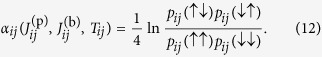


Note that the assumption that 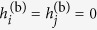 implies that *p*_*ij*_(↑↑) = *p*_*ij*_(↓↓) and *p*_*ij*_(↑↓) = *p*_*ij*_(↓↑), and that in this case (12) reduces to (7).

### Benchmark studies

To generate a single instance with range *r*, the local fields {*h*_*i*_} were uniformly set to zero, and each available *J*_*ij*_ was independently and uniformly selected from {−*r*, −*r* + 1, …, −1, 0, 1, …, *r*}. The resulting instance was then scaled by the overall factor 0.9/*r* so that the largest magnitude |*J*_*ij*_| was 0.9. (This was necessary, rather than scaling to 1 as in previous studies, to allow for consistency with future experiments in which the *J* biases are corrected.) 100 such instances were generated and run twice with 1,000 annealing cycles for each of the same (uniformly randomly generated) 10 gauges. In all runs, {*J*_*ij*_} were programmed as in the instances. For the first set of runs, which we call “uncorrected”, the {*h*_*i*_} were also programmed as in the instances, i.e. to zero. For the other set, which we call “*h*-corrected”, the local fields were programmed to the inverse of the biases computed via experiments as in Eq. 4.

#### Greedy and elite mean metrics

The greedy comparison is as follows: the energies of all states returned were computed, and those for all gauges were grouped together. Whichever method (uncorrected or corrected) returned the lower minimum energy was deemed to have performed better. If the minimum energies were the same, the tie was broken by the number of times that energy was returned. If this number was the same, the method with the second-lowest energy was deemed to have performed better, with ties broken by the number of times the second-lowest energy was returned, and so on. The “elite mean” score function^23^, a quantity previously introduced to allow comparison of the performance of different programming parameters in quantum annealers when the success probabilities are too low (and thus noisy), is defined as the mean energy of the “elite” states, i.e. those with the lowest energies. The elite mean is parameterized by the fraction of energies over which to take the mean; here we use 2%. For *r* = 1 and *r* = 2, there were 6 and 2 instances, respectively, for which the elite mean comparison was tied, all but one due to success probabilities greater than 2% for both the corrected and uncorrected experiments.

## Additional Information

**How to cite this article**: Perdomo-Ortiz, A. *et al*. Determination and correction of persistent biases in quantum annealers. *Sci. Rep*. **6**, 18628; doi: 10.1038/srep18628 (2016).

## Supplementary Material

Supplementary Information

## Figures and Tables

**Table 1:** Comparison of performance with and with *h*-correction on benchmarks.

Range *r*_*J*_	1	2	4	8	16
Greedy	0.58	0.63	0.59	0.68	0.53
Elite mean	0.65	0.65	0.73	0.72	0.67

The probability that correcting for *h* biases (using data from a single experiment) improved performance on 100 random instances from an ensemble parameterized by the range of values *r*. Performance was compared according to two metrics: greedy comparison of the energies and degeneracies, and comparison of the elite mean score function.
