# Supplementary material for: Determination and correction of persistent biases in quantum annealers
Source: Sci Rep. 2016 Jan 19;6:18628. doi: 10.1038/srep18628 (PMC4725997; doi:10.1038/srep18628)
Supplement: Supplementary Information [file srep18628-s1.pdf]

Supplementary Information: Determination and correction of persistent biases in quantum annealers

Alejandro Perdomo-Ortiz,[1,2,*] Bryan O'Gorman,[1,3] Joseph Fluegemann,[1,4] Rupak Biswas,[5] and Vadim N. Smelyanskiy[6]

[1] *Quantum Artificial Intelligence Lab., NASA Ames Research Center, Moffett Field, CA 94035, USA*
[2] *University of California Santa Cruz at NASA Ames Research Center, Moffett Field, CA 94035, USA*
[3] *SGT Inc., 7701 Greenbelt Rd, Suite 400, Greenbelt, MD 20770, USA*
[4] *San Jose State Research Foundation at NASA Ames Research Center, Moffett Field, CA 94035, USA*
[5] *Exploration Technology Directorate, NASA Ames Research Center, Moffett Field, CA 94035*
[6] *Google, 150 Main St, Venice Beach, CA, 90291*

(Dated: December 13, 2015)

I. QUANTUM ANNEALING

A. Computational problem

QA is designed to solve the classical problem of finding the ground state of an Ising system, i.e. finding an assignment of a set of n classical spins $\mathbf{s} = (s_i)_{i=1}^n \in \{\pm 1\}^n$ that minimizes the following energy function,

$$E(\mathbf{s}) = \sum_{1 \le i \le n} h_i s_i + \sum_{1 \le i < j \le n} J_{ij} s_i s_J. \tag{1}$$

Here, the set of real-value parameters $\{h_i\}$ and $\{J_{ij}\}$ define the problem instance. This Ising problem is an NP-hard [1].

B. Physical process

Quantum Annealing (QA) is a metaheuristic for solving combinatorial optimization problems [2]. The main procedure is this: a final Hamiltonian is constructed for some set of qubits such that its ground state encodes the optimal solution to the desired problem, those qubits are prepared in the ground state of an initial Hamiltonian, and then over the course of the annealing process the initial Hamiltonian is continuously transformed into the final Hamiltonian. While there is ambiguity regarding the exact definition of the term "QA" and its differences with its closely related spin-off, adiabatic quantum computation[3], we define it here to allow for nonadiabiticity in the annealing process and finite temperature, on the latter of which our methods are based.

To solve a computational problem, a Hamiltonian is constructed whose ground state encodes the optimal solution of the given problem,

$$H_{\text{final}} = \sum_{1 \le i \le n} h_i \sigma_i^{(z)} + \sum_{1 \le i < j \le n} J_{ij} \sigma_i^{(z)} \sigma_j^{(z)}, \tag{2}$$

i.e. the quantum analog of (1). The system is initialized in the easily-prepared ground state of another Hamiltonian, usually

$$H_{\text{init}} = \sum_{1 \le i \le n} \sigma_i^{(x)}, \tag{3}$$

whose ground state is a uniform superposition of all 2^n computational basis states. Then the Hamiltonian is slowly changed from the former to the latter; explicitly,

$$H(t) = A(t) H_{\text{init}} + B(t) H_{\text{final}}, \tag{4}$$

where $A(t), B(t) \ge 0$ define the "annealing profile" and are such that $A(0), B(t_{\text{anneal}}) >> 0$ and $A(T) = B(0) = 0$, where t_{anneal} is the annealing time.

C. D-Wave devices

D-Wave devices consist of an array of coupled superconducting flux qubits that is effectively a Ising spin system with programmable spin-spin couplings and local fields, longitudinal and transverse. The qubits are arranged in a so-called Chimera topology consisting of a square lattice array of bipartite unit cells. The two devices used in this work consist of 512 nominal qubits in an 8-by-8 array of 8-qubit unit cells. Post-fabrication testing indicated that only 509 and 424 qubits were usable in the NASA and D-Wave devices, respectively. In both devices, the programmed values of $\{h_i\}$ and $\{J_{ij}\}$ are specified by unitless parameters in $[-2, 2]$ and $[-1, 1]$, respectively, where 1.0 corresponds to an actual energy of 3.2 GHz. Further details of the hardware can be found in [4]. Thoughout this paper, we use the standard 1-based indexing for the qubits of a D-Wave device with 512 qubits, as in, e.g., Fig. 7(b) of [5]. (While the set of functional qubits differs between actual devices, the convention is to still assign the indices to all qubits and to disregard the broken ones.)

* Corresponding author's e-mail: alejandro.perdomoortiz@nasa.gov

II. INTRINSIC NOISE LIMIT

Figures S1(a,b,d,e) shows the probability $p_i^{(r)} = p_i^{(r)}\big(\downarrow |h_i^{(\mathrm{p})} = 0.1\big)$ for each of the qubits in two different unit cells and for each of the 100 runs. From this we see two fundamental phenomena: the run-to-run fluctuation of the probability $p_i^{(r)}$, and the presence of some qubits for which the typical probability is well separated from the others, to a degree greater than the inherent variability. The latter indicates the presence of the qubit-specific biases that we hope to determine and correct, while the former provides a limit to the precision with which we can control the effective h_i.

To compare the variability of $\{p_i(h^{(\mathrm{p})})\}$ over different values of $h^{(\mathrm{p})}$, for each qubit i and value of $h^{(\mathrm{p})}$ we compute the 100 values of $\tilde{\tilde{h}}_i^{(r)} = \alpha(p_i^{(r)}(h^{(\mathrm{p})}))\tilde{T}^{(h)}$ from the 100 runs. Figure S1(e) shows the standard deviation $\sigma_{\tilde{\tilde{h}}_{55}^{(r)}}$ of $\{\tilde{\tilde{h}}_{55}^{(r)}\}$ over the 100 runs; the plot is typical. Importantly, the standard deviation $\sigma_{\tilde{\tilde{h}}_i^{(r)}}$ is seemingly independent of the programmed $h_i^{(\mathrm{p})}$ for the values of $h^{(\mathrm{p})}$ considered. To characterize the typical variance of h_i, for each qubit the mean $\bar{\sigma}_{\tilde{\tilde{h}}_i}$ of standard deviations $\{\sigma_{\tilde{\tilde{h}}_i^{(r)}}\}$ was taken over the values of $h^{(\mathrm{p})}$. Figure S1(f) shows a histogram of this mean standard deviation over all of the qubits. The distribution is quite tight, with an average of $\bar{\bar{\sigma}}_{\tilde{\tilde{h}}} = 0.0156$. This run-to-run variation in the estimate of h_i for a given $h^{(\mathrm{p})}$ ultimately leads to a limit on the precision with which we can estimate $h_i^{(\mathrm{b})}$, though quantitatively the exact limit depends on the number of runs, number of reads per run, and values and number of $h^{(\mathrm{p})}$s examined.

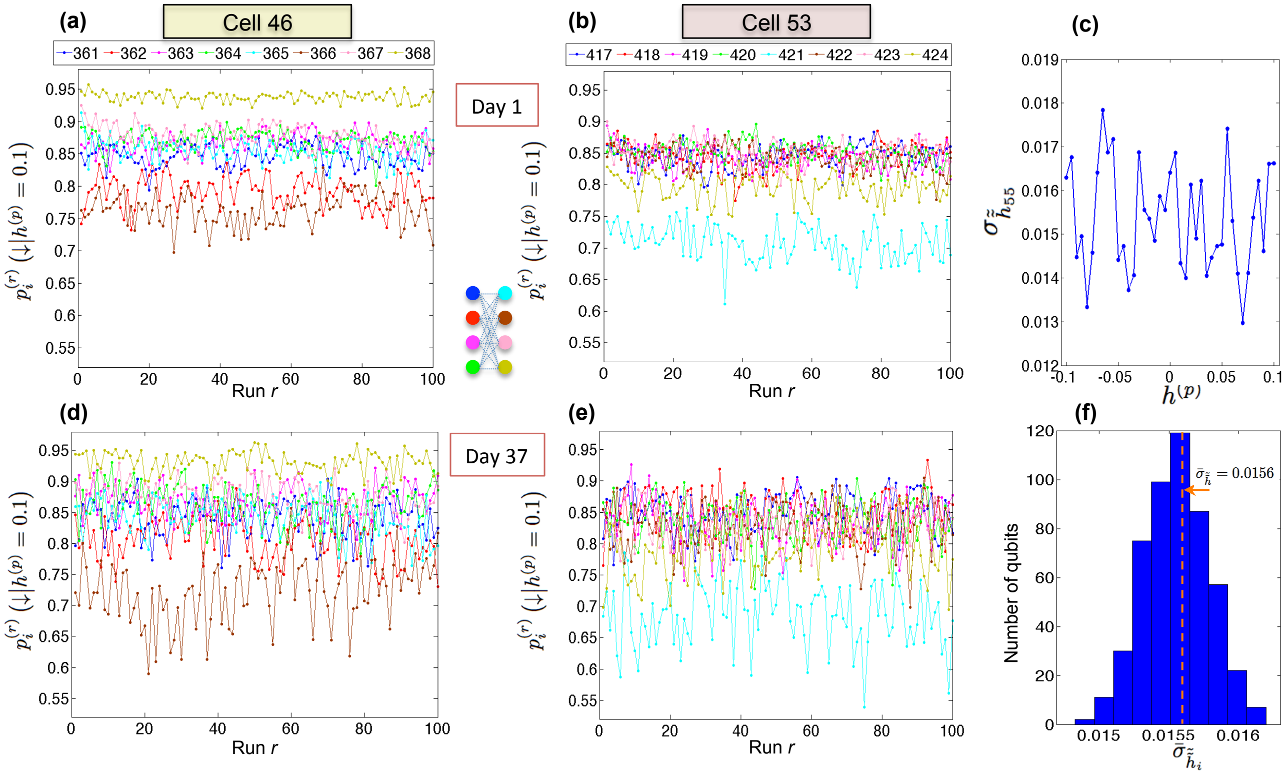

Figure S1. Persistent systematic biases and inherent h_i noise limit. (a,b,d,e) The probability $p_i^{(r)}\big(\downarrow |h^{(\mathrm{p})} = 0.1\big)$ of qubit i being spin-down in the 100 runs $\{r\}$ of 1000 reads each, for two experiments on the 16 qubits of two adjacent unit cells, done 36 days apart and in which the programmed value of all the qubits was $h^{(\mathrm{p})} = 0.1$. Two important aspects are apparent that lay the foundation for the present work: there are qubits whose deviation from the rest is much greater than the noise level, and that this deviation persists for more than a month. As we show, these deviations in the probabilities are indicative of biases in the programmable parameters. (c) For a single, typical qubit, 55, the standard deviation over 100 runs of the estimates $\{\tilde{\tilde{h}}_{55}^{(r)}\}$ of h_{55} versus the programmed $h^{(p)}$. (f) The means $\{\bar{\sigma}_{\tilde{\tilde{h}}_i}\}$ over the programmed values $\{h^{(p)}\}$ of the standard deviations $\{\sigma_{\tilde{\tilde{h}}_i}\}$, as in (c). The values of $\tilde{\tilde{h}}_i$ in (c,f) were calculated using the mean temperature $\tilde{T}^{(h)}$.

III. QUBIT TEMPERATURES

Figure S2(a) shows the distribution of estimated "qubit temperatures" $\{\tilde{T}_i\}$. Recall that each qubit temperature T_i extracted a linear fits of the points $\{(h_i^{(\mathrm{p})}, \tilde{\alpha}_i)\}$, e.g. Fig.1(c) in the main text. That the variance of the temperatures of the qubits is as large as indicated here seems unlikely. For that reason, a more physically plausible "device temperature" was estimated, i.e. $\tilde{T}$ or $\bar{T}$, as described in the Methods section in the main text. Data indicate that the variance is partly systematic, i.e. not due to the fluctuation of the experimental values on which the estimates is based.

Figure S2(b) shows, for three related experiments on the NASA device, the standard deviation $\sigma_{\tilde{\tilde{h}}_i}$ over the qubits of the estimated value $\{\tilde{\tilde{h}}_i\}$ for different values of $h^{(\mathrm{p})}$. One experiment was without any correction; the other two used corrections calculated in two different ways, one using the qubit temperatures $\{\tilde{T}_i\}$ and the other the median temperature $\tilde{T}^{(h)}$. Both corrections clearly improve the variance, but the similarity of the degree of improvement justifies our use of an effective device temperature.

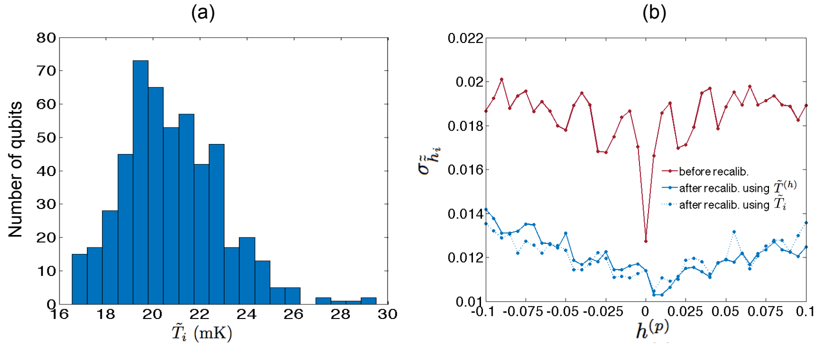

Figure S2. Qubit temperatures and their effect. (a) The estimates of the individual qubit temperatures $\{\tilde{T}_i\}$ calculated from a single experiment without correction. (b) The standard deviation $\sigma_{\tilde{\tilde{h}}_i}$ of the estimated field values $\tilde{\tilde{h}}_i$ for different values of $h^{(\mathrm{p})}$.

IV. PERSISTENCY OF THE BIASES

Fig. S3 shows the correlation of the biases $\{\tilde{\tilde{h}}_i^{(b,1)}\}$ as estimated from single experiments repeated at different times. Each data point corresponds to the bias in each of the 509 qubit of the NASA device. All experiments were performed as described in the Methods section in the main text, with 100 runs of 1000 annealing cycles each for 41 evenly spaced values of $h^{(\mathrm{p})}$ in [-0.1,0.1]. Notice the strong correlation for intervals greater than one month. The experimentally determined median temperature $\tilde{T}^{(h)}$ can also be considered constant, with values within 1% from the average of these four realizations: $\tilde{T}^{(h)} = 19.0, 19.2, 19.2,$ and 19.2 mK, for the experiments on 10-09-2014 at 14:00, on 10-09-2014 at 16:30, on 10-24-2014 at 11:15, and on 11-12-2014 at 7:45, respectively.

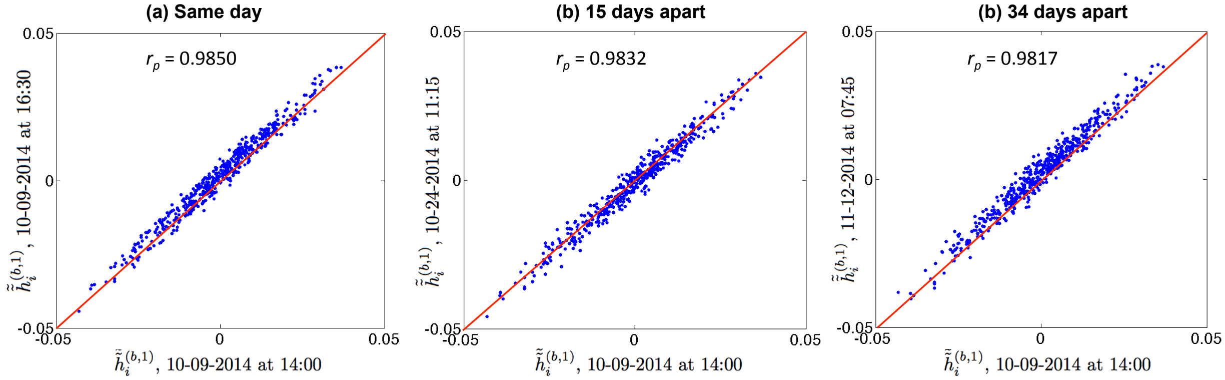

Figure S3. Persistency of systematic biases for time intervals.

[1] Barahona, F. On the computational complexity of ising spin glass models. Journal of Physics A: Mathematical and General **15**, 3241 (1982).

[2] Kadowaki, T. & Nishimori, H. Quantum annealing in the transverse ising model. Phys. Rev. E. **58**, 5355 (1998).

[3] Farhi, E. et al. A quantum adiabatic evolution algorithm applied to random instances of an NP-Complete problem. Science **292**, 472–475 (2001).

[4] Harris, R. et al. Experimental demonstration of a robust and scalable flux qubit. Phys. Rev. B **81**, 134510 (2010).

[5] Pudenz, K. L., Albash, T. & Lidar, D. A. Error-corrected quantum annealing with hundreds of qubits. Nat Commun **5** (2014).
